# Electrochemical Detection of Different Foodborne Bacteria for Point-of-Care Applications

**DOI:** 10.3390/bios13060641

**Published:** 2023-06-12

**Authors:** Tailin Wu, Ajay Kumar Yagati, Junhong Min

**Affiliations:** School of Integrative Engineering, Chung-Ang University, Seoul 06974, Republic of Korea; tailin@cau.ac.kr (T.W.); yagati@cau.ac.kr (A.K.Y.)

**Keywords:** bacteria, electrochemical, aptamer, sensor, DPV, POC testing

## Abstract

Bacterial infections resulting from foodborne pathogenic bacteria cause millions of infections that greatly threaten human health and are one of the leading causes of mortality around the world. To counter this, the early, rapid, and accurate detection of bacterial infections is very important to address serious health issue concerns. We, therefore, present an electrochemical biosensor based on aptamers that selectively bind with the DNA of specific bacteria for the accurate and rapid detection of various foodborne bacteria for the selective determination of bacterial infection types. Different aptamers were synthesized and immobilized on Au electrodes for selective bindings of different types of bacterial DNA (Escherichia coli, Salmonella enterica, and Staphylococcus aureus) for the accurate detection and quantification of bacterial concentrations from 10^1^ to 10^7^ CFU/mL without using any labeling methods. Under optimized conditions, the sensor showed a good response to the various concentrations of bacteria, and a robust calibration curve was obtained. The sensor could detect the bacterial concentration at meager quantities and possessed an LOD of 4.2 × 10^1^, 6.1 × 10^1^, and 4.4 × 10^1^ CFU/mL for S. Typhimurium, E. Coli, and S. aureus, respectively, with a linear range from 10^0^ to 10^4^ CFU/mL for the total bacteria probe and 10^0^ to 10^3^ CFU/mL for individual probes, respectively. The proposed biosensor is simple and rapid and has shown a good response to bacterial DNA detections and thus can be applied in clinical applications and food safety monitoring.

## 1. Introduction

The rapid qualitative and quantitative detection of bacterial infections remains a challenging and important issue for clinical diagnosis, environmental monitoring, and food safety applications [1]. With communicable diseases being highlighted as a global health priority and increasing mortality rates due to bacterial infections, early detection, prevention, and effective treatment of infections become crucial [2]. Further, antimicrobial susceptibility testing (AST) is essential for treating many types of bacterial infections as antimicrobial resistance (AMR) is becoming a worldwide problem [3]. As the World Health Organization identified AMR as a global threat, many AST studies have been conducted to address the solution for the informed use of antibiotic doses. Many techniques such as the polymerase chain reaction [4], mass spectroscopy [5] microarrays [6], microfluidics [7], and impedance-based [8] approaches were aimed at bacterial growth monitoring characteristics, and drug candidate evaluations at a reduced time response. The traditional culture method, being the gold standard approach for bacteria detection, is limited due to being laborious and time-consuming, and has chances of false-positive signals caused by uncultivated bacteria [9]. Conventional techniques such as a polymerase chain reaction [10] or enzyme-linked immunosorbent assays used for bacterial pathogens require modern laboratories and skilled personnel [11]. Therefore, new technologies which are suitable for the routine and rapid identification of bacterial infections are of the utmost priority to reduce the risk to public health and control the spread of infections.

Over the past two decades, many attempts have been made to adopt biosensor technology as a point-of-care testing (POC) tool [12]. Electrochemical biosensors are most suitable for POC technologies due to their fast response, portability, and low cost [13,14,15]. In addition, biosensors simplify the sample preparation steps and allow the detection of a broad spectrum of analytes in complex biological matrices [16,17]. While the recent emergence of nucleic acid (NA)-based biosensing technologies has revolutionized viral or bacterial diagnoses due to their high thermal stability and low immunogenicity [2,18], until that point, even for preliminary test results, it was entirely dependent on the enzyme-linked immunosorbent (ELISA) assays [19]. Aptamers are small oligonucleotide sequences that are engineered to bind with specific target molecules with high affinity [20]. These short nucleotide sequences can bind through electrostatic [21], hydrophobic interactions [22], or hybridization with complementary sequences [23], resulting in binding almost any target and thus possessing a wide variety of applications. Most of these aptamers were tethered with some specific functional groups [24] to bind with various types of modified or bare electrode surfaces [25]. Thus, these NA-based biosensors mostly utilize the hybridization of nucleotide probes to bind with their target sequence, i.e., the analyte of interest for recognition in various transducing mechanisms such as optical [26,27], electrochemical [28,29], and quartz crystal microbalance-based biosensors [30].

Therefore, in this study, an electrochemical biosensor for the rapid detection of various types of bacteria using Au disk electrodes was proposed without using any labels. Selective aptamers were synthesized and used as probes that were immobilized on Au electrodes, and a common probe that binds with all types of bacteria was evaluated. Different concentrations of bacterial deoxyribonucleic acid (DNA) were allowed to bind with the immobilized probe, and a quantification was made by generating a calibration curve. The sensor’s cross-reactivity was estimated by evaluating the binding reaction of bacterial DNA with different probes. Here, no labeling methods or any surface modification of Au electrodes were made, which improve the sensitivity at the cost of reproducibility of the sensor. Thus, the proposed strategy is very simple, portable, and rapid and can be a universally acceptable multiplexing detection approach for the detection of pathogenic bacteria for a broad range of applications including public health and early diagnosis.

## 2. Materials and Methods

### 2.1. Bacterial Culture Preparation

Using the Luria-Bertani (LB) broth medium, the target foodborne pathogens *E. coli*, *S. aureus*, and *S. Typhimurium* were cultured by shaking in an oscillator at 37 °C and 180 rpm for 12 h. Bacterial DNA was extracted by using a DNA mini kit, and its concentration was measured by nanodrop spectrophotometry (ThermoFisher Scientific) and stored at −20 °C. The concentration of the bacterial stock solution was determined by the plate method. The extracted ds-DNA solution was sequentially diluted to the desired concentration and heated at 95 °C for 1.5 min using a heating block to prepare the ss-DNA solution for storage. All pathogen preparation procedures were carried out in a biosafety cabinet, and all waste was inactivated before disposal.

### 2.2. Apparatus and Instrumentation

All voltametric measurements were carried out using CHI 660E potentiostat (CH Instruments, TX, USA) in a three-electrode configuration. The patterned Au electrodes served as working, counter, and pseudo reference electrodes, respectively. The potential window for all differential pulse voltammetry was chosen from 0.4 to −0.7 V. The measurements were carried out in an electrolyte solution composed of 5 mM potassium ferricyanide/ferrocyanide ([Fe(CN)_6_]^3−/4−^) in 0.1 M potassium chloride (KCl), both purchased from Merck.

### 2.3. Aptamer Probe Preparations

**Figure 1:**
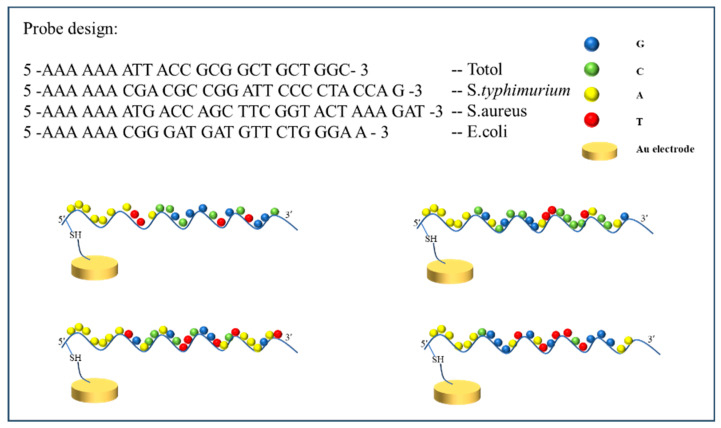
Schematic diagram shows the selection of probes used in the detection of bacterial DNA types along with their immobilization on the Au electrode surface.

Aptamers are custom-designed to selectively bind with the DNA of *Escherichia coli*, *Salmonella typhimurium*, and *Staphylococcus aureus*, and an aptamer for total bacteria detections as mentioned below (Figure 1) was purchased from Genotech, Daejeon, South Korea.

To prepare the DNA probe for immobilization on the Au electrode surface, under optimized conditions, a concentration of 1 mM was prepared. Next, the diluted DNA probe was mixed with 10 mM Tris(2-carboxyethyl)phosphine hydrochloride (TCEP-HCl, Thermo Scientific™, Waltham, MA, USA) in the dark and incubated for 1 h. This step is crucial for reducing the disulfide bonds present in the thiol-modified aptamer end.

### 2.4. Gold (Au)-Disk Electrode for Aptamer Immobilization and Bacteria Detection

**Figure 2:**
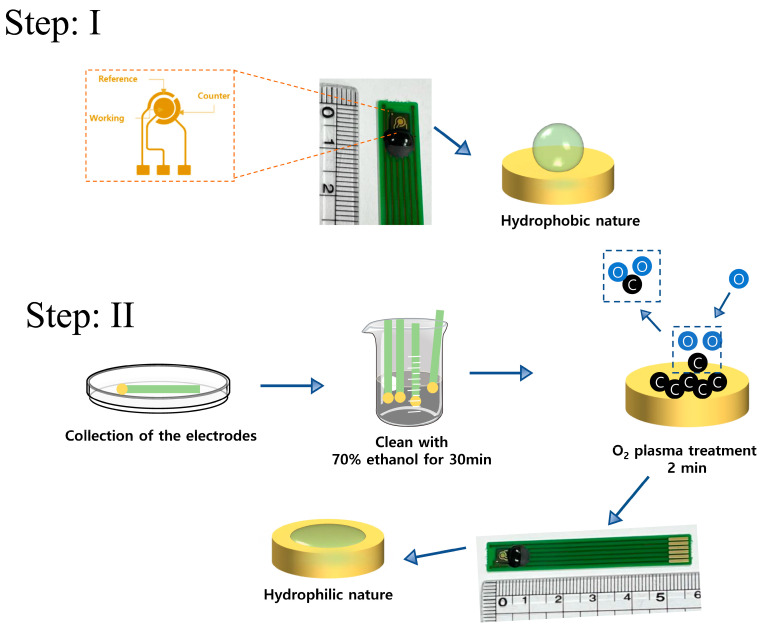
Schematic diagram for the electrode and activation process. Step I: the electrode surface was hydrophobic; in Step II: the plasma treatment was performed to make the surface hydrophilic.

Au electrodes were prepared in a three-electrode configuration with Au disks acting as working electrodes and Au rings as counter and reference electrodes as shown in Figure 2. To prepare the Au electrodes for experiments, initially, a pre-treatment process was performed. Firstly, the screen-printed electrode was cleaned with a 70% ethanol solution. Then, the electrode surface was washed with ultra-pure water to ensure a clean and impurity-free surface. Afterward, nitrogen gas was used to dry the surface and ensure it was free of moisture. Finally, O_2_ plasma treatment was performed to make the gold electrode surface become hydrophilic and contamination-free for its use in subsequent experiments.

### 2.5. Construction of Electrochemical Biosensor for Different Types of Bacteria Detection

**Figure 3:**
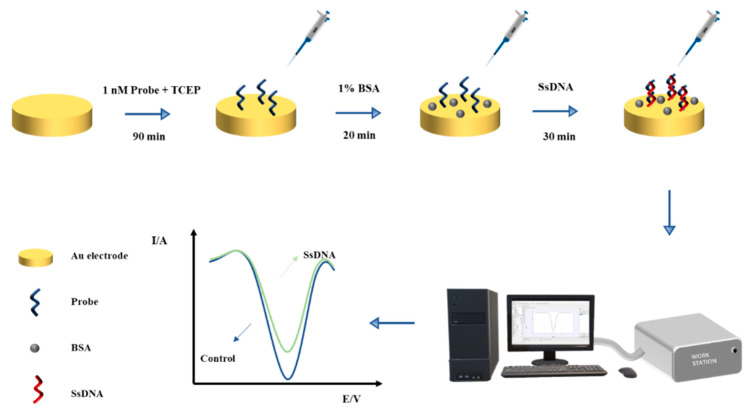
Schematic diagram for the formation of the detection platform. A detection workflow shows the step-by-step modifications performed on the Au electrode surface for pathogenic bacterial detection.

To construct the aptamer-based biosensor for bacteria detection, at first, 2 μM of different types of the aptamer probe solution was dissolved in a 10 mM TCEP mixture and incubated for 1 h at room temperature in the dark. This step was crucial for reducing the disulfide bonds present in the thiol-modified adapter end. Subsequently, 20 μL of the aptamer probe was dropped onto the surface of the Au electrode at 25 °C, and after 1.5 h of incubation, for immobilization of the Au electrode through disulfide bonds (Au-S). Subsequently, the electrode was washed 3 to 4 times with the wash buffer, and 1% BSA (*w*/*v*) was added to the electrode, which was then incubated in the dark at room temperature for 20 min to block the non-specific sites on the electrode surface (Figure 3). The electrode surface was then washed with ultra-pure water and dried with nitrogen gas. After each modification step, the electrode was thoroughly washed with a wash buffer. Electrochemical detection was performed using differential pulse voltammetry (DPV), and the potential range of the DPV measurement was selected from −0.1 to 0.7 V, with a pulse amplitude of 10 mV, and a pulse cycle of 0.5 s.

### 2.6. Optimization of Conditions for Sensitive Electrochemical Sensor

To achieve optimal analytical performance, the experimental conditions of the electrochemical biosensor were optimized. Firstly, different concentrations of the probe analyte aptamer (0.5, 1, 1.5, and 2 μM) were selected and added onto the surface of the Au electrode after TCEP treatment to determine the optimal reaction signal. After determining the optimal aptamer concentration, experiments for incubation time were carried out as 0.5, 1, 1.5, 3, and 6 h, respectively. In addition, different concentrations of BSA (1%, 2%, and 3%) were selected and added onto the surface of the Au electrode for detection, to determine the optimal reaction signal. Furthermore, for the optimization of the BSA incubation time, detection reactions were carried out at 10, 20, 30, 60, and 120 min. Finally, the thermal denaturation temperature of the double-stranded DNA was optimized by selecting different temperature conditions (75 to 95 °C, with an increment of 5 °C) for denaturing the ds-DNA into ss-DNA, with different heating times (0.5, 1, 1.5, 2, 2.5, and 3 min), to determine the optimal reaction signal. In all of the above condition optimizations, the target analytes *Escherichia coli*, *Salmonella typhimurium*, and *Staphylococcus aureus* DNA had a concentration of 10^4^ CFU/mL and were subjected to three repeat experiments.

### 2.7. Sensitivity and Selectivity Detection

For the sensitivity analysis, at first, the bacteria concentration was determined using a nanodrop spectrophotometer. Next, target concentrations of 10^1^ to 10^7^ CFU/mL, as well as a buffer control group, were achieved by diluting the bacterial solution with a 10-fold concentration gradient. Under optimal reaction conditions, DPV measurements were performed to analyze the bacterial DNA bindings with different aptamer probes. The detection was repeated three times to ensure the reliability of the experimental results.

## 3. Results and Discussion

### 3.1. Electrochemical Biosensing Assay Principle

The detection principle of this experiment involved the combination of biomolecular recognition and electrochemical sensing to achieve the high-sensitivity detection of target pathogenic bacterial DNA molecules. An electrochemical biosensor was designed to rapidly detect various foodborne pathogenic bacteria (Figure 1). Here, an aptamer was used as a molecular probe to recognize pathogenic bacteria such as *E. coli*, *Salmonella*, and *Staphylococcus*, and was stably bound to the Au electrode surface after thiol modification. Compared to antibodies, aptamers have advantages such as simple and rapid synthesis, high affinity, good stability, flexible modification, resistance to denaturation, and low cost. Based on the aptamer-based electrochemical immunoassay, which can avoid the limitations of multiple antibodies, the specific detection of pathogenic bacteria has been widely applied. Therefore, we established an aptamer-based electrochemical biosensor for foodborne pathogenic bacteria. Selective aptamer probes were immobilized on the Au electrode surface vis sulfur-gold chemistry and then hybridized to bacterial DNA as in an antigen–antibody interaction (Table 1). With the additions of different concentrations of bacterial DNA, and in the presence of the redox marker ([FeCN_6_]^3−/4−^), the ions approached the electrode surface due to the bindings of the bacterial DNA, causing a hindrance of electron transfer between ferro/ferri cyanide ([Fe(CN)_6_]^3−/4−^) ions, resulting in a significant decrease in the peak value of differential pulse voltammetry (DPV) leading to a clear change in the electrochemical signal. The measurements were performed using a specific aptamer probe as well as a common probe that binds with any kind of bacterial DNA.

### 3.2. Optimization of Different Experimental Conditions

To achieve the best detection sensitivity of the proposed method, we optimized different detection parameters, such as aptamer concentration for target capture, aptamer incubation time, BSA concentration for blocking, BSA incubation time, the thermal denaturation temperature of double-stranded DNA, and heating time. The concentration of target pathogenic bacteria used in the experiments was 10^2^ CFU/mL. The concentration of the aptamer for capturing the target had a significant impact on the overall response of the electrochemical aptasensor. When the concentration is too low, it cannot effectively capture and identify the target pathogenic bacteria. Theoretically, the larger the aptamer with a target capture function, the larger change in the electrochemical signal was expected to be displayed. However, when the amount of aptamer immobilized on the Au electrode surface is too high, the spatial hindrance and electrostatic repulsion between the nucleic acid and aptamer will hinder the electron transfer at the electrode surface, thus reducing the sensitivity of the biosensor. To confirm our hypothesis, the detection performance of the biosensor was initially evaluated by optimizing the binding probe concentration. Different concentrations of aptamer probes of 0.05, 0.1, 0.5, 1, and 5 nM were allowed to bind with the Au electrode surface and were quantified using differential pulse voltammetry (DPV).

**Figure 4:**
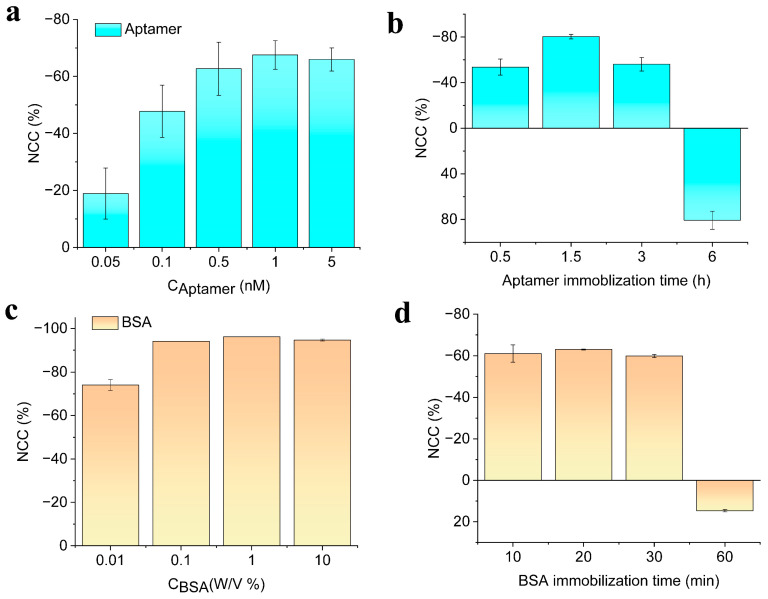
Biosensor optimizations for (**a**) various concentrations of receptor aptamer probe; (**b**) immobilization time; (**c**) time of BSA immobilization; and (**d**) concentration of BSA optimizations. All measurements were taken in triplicate, and the error bars represent standard deviations for individual measurements (*n* = 3).

The DPV curves demonstrated that the immobilization of the aptamer probe showed an increment in the current with increasing concentration up to 1 nM (Figure 4a), and this optimum concentration was thus applied for further experiments. By increasing the concentration of the probe above 1 nM, the electrochemical signal reached a saturation value. In addition, the time required for the binding process with the underlying electrode surface was then optimized. As can be seen in Figure 4b, after incubating the aptamer strands on the Au electrode for a duration of 0.5, 1.5, 3, and 6 h, the DPV curves were measured, and it was found that the immobilization time of 1.5 h possessed the highest change (decrement in the current) concerning the other concentrations. It was evaluated by measuring the normalized current change (NCC %) as

(1) $$\mathrm{NCC}\;\%=\frac{I_{a}-I_{control}}{I_{control}}*\;100\%$$

where *I*_a_ is the DPV current measured after binding, and *I*_control_ is the current measurement before binding.

Therefore, an aptamer probe with 1 nM for 1.5 h immobilization time was adopted for further experimental procedures. Subsequently, the bovine serum albumin (BSA) which was used as non-specific blocking was also optimized for its blocking concentration and time of immobilization as shown in Figure 4c,d. After the bacterial DNA hybridized with the aptamer probe, the concentration of BSA from 0.01, 0.1, 1, and 10% was incubated, and the electrochemical signal showed a better response for the concentration of 1%, while excess concentration did not show any significant change in the overall sensitivity of the electrochemical sensor. Thus 1% BSA was chosen for further studies, and this concentration was immobilized on the fabricated sensor surface for the duration of 10, 20, 30, and 60 min. An excess incubation time of BSA beyond 20 min did not show any change in the EC signal and was sufficient to block the surface of the electrode sensor, thus 1% BSA for an incubation time of 20 min was the subsequent experimental analysis of bacterial DNA detection.

#### Effect of Temperature, Heating Time, and Sonication Time on DNA Hybridization Detection

**Figure 5:**
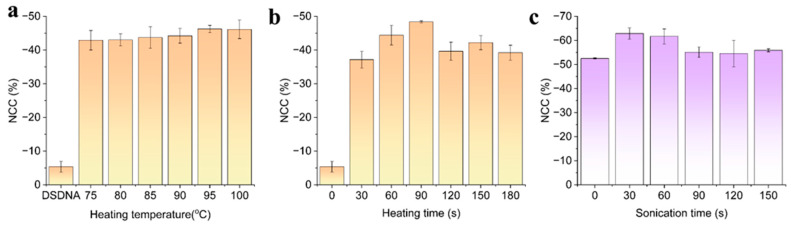
DPV response observed from the extraction of ssDNA from the sonication method under different optimization conditions such as (**a**) heating temperature; (**b**) heating time; and (**c**) sonication time, respectively.

Another crucial factor that influences the proper quantification of bacterial pathogens is binding efficiency. To achieve this, the proper extraction of single-stranded DNA (ss-DNA) for effective bindings with aptamer probes is imminent. Thus, in another experiment, optimal conditions for extracting single-stranded DNA from the bacteria which is double-stranded were studied. These studies included the influence of temperature, heating time, and sonication time as shown in Figure 5a−c. Heating temperatures ranging from 75 to 100 °C with an increment of 5 °C were used to denature the ds-DNA of bacteria and subjected to binding with the aptamer probe. From the results, it was found that 90 °C was optimum for the formation of ssDNA and efficient bindings; we also tested the required heating time starting from 30 to 180 s and found that 90 s was sufficient to generate ssDNA of pathogenic bacteria. Subsequently, the sonication method of extracting the ssDNA by sonicating the dsDNA samples for various sonication times for 30, 60, 90, 120, and 150 s was applied to the sensor surface by dropping a few microliters of DNA on the electrode surface. DPV responses for the immobilization performed with these sonication times also revealed that 30 s of sonication time was enough to separate the DNA molecules. Thus, by optimizing these conditions, we performed further steps in the formation biosensing assay and proceeded with the detection of bacterial pathogens.

### 3.3. Characterizing the Biosensing Surface for Bacterial Pathogen Detection

**Figure 6:**
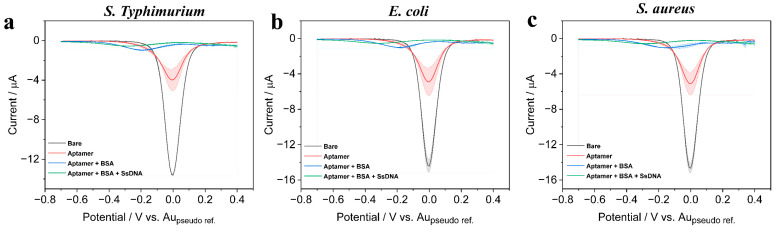
DPV response observed for step-by-step modifications performed on the sensor along with three types of pathogenic bacterial DNA bindings.

The fabrication steps of the biosensor were illustrated as shown in Figure 1, which shows the step-by-step modifications on the Au electrode surface. These modifications were characterized with DPV by scanning in a potential window ranging from 0.4 to −0.7 V for each modification performed on the Au surface (Figure 6). To start with, clean Au electrodes were immobilized with aptamer probes through Au-S bond formation; subsequently, the BSA was immobilized to avoid non-specific bindings; and then the three types of bacterial DNA were allowed to bind with the receptor surface. We conducted electrochemical DPV tests in an electrolyte containing 1 mM K_3_Fe(CN)_6_, 1 mM K_4_Fe(CN)_6_, and 0.1 M KCl solution to test each step of the sensor assembly process. The results showed that the transfer rate of [Fe(CN)_6_]^3−^ and [Fe(CN)_6_]^4−^ ions on the bare electrode surface was not significantly hindered, resulting in the maximum peak of DPV. However, when thiolated DNA reacted with the electrode surface, a large amount of thiolated DNA occupied the electrode surface, hindering the electron transfer, and resulting in a significant decrease in the DPV peak. Then, adding 1% BSA to block the non-specific recognition sites on the electrode surface again hindered the electron transfer, resulting in a decrease in DPV peak. After adding the target SsDNA, the DNA probe was base-paired with ssDNA gene bases, further reducing the electron transfer rate on the electrode surface and resulting in a decrease in DPV peak. As can be seen in Figure 6a−c, the bare electrode showed the maximum current response as there was no obstruction to the [FeCN_6_]^3−/4−^ ions to move freely towards the electrode and undergo the redox reactions; however, upon subsequent modifications with aptamers and their bindings, the DPV response showed a significant decrement in the current level due to the inhibition of [FeCN_6_]^3−/4−^ ions to move towards the electrode to participate in redox reactions. These DPV test results validated the step-by-step assembly process of the electrochemical biosensor on the screen-printed gold electrode surface and demonstrated the feasibility of the proposed approach.

### 3.4. Analytical Performance of the Sensor for Quantification of Bacterial Types with DPV

**Figure 7:**
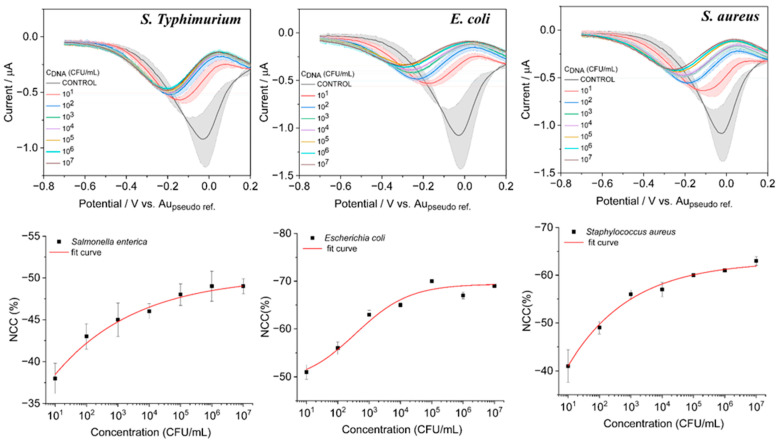
DPV response observed from the sensor for different bacterial DNA for concentrations of 10^1^ to 10^7^ when incubated with total bacteria probe which has selectivity for all types of bacteria.

**Figure 8:**
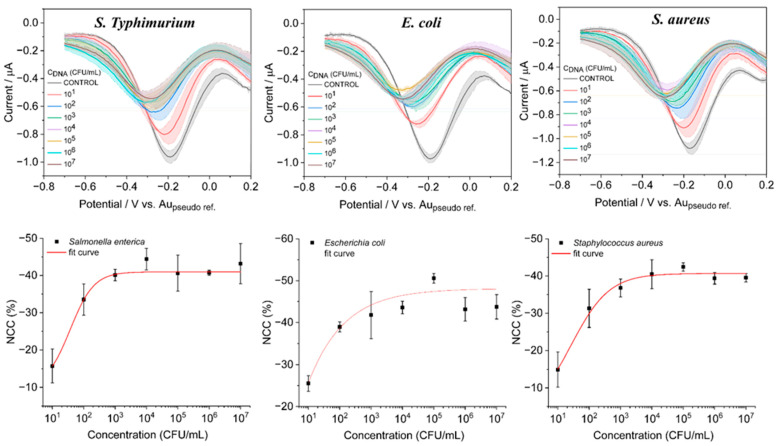
DPV response observed from the sensor for different bacterial DNA for concentrations of 10^1^ to 10^7^ when incubated with a specific receptor probe that has selectivity towards particular bacteria.

The analytical performance of the sensor towards the detection of various bacterial DNA concentrations was evaluated by conducting DPV experiments. All three variants of bacterial DNA ranging from 10^1^ to 10^7^ CFU/mL were allowed to conjugate with the biosensing surface that consisted of an aptamer probe which can detect all the variants of bacterial DNA as shown in Figure 7, and specific aptamer probes (Figure 8). As can be seen from the responses of bindings of different concentrations of DNA with both types of aptamer probes, the DPV currents gradually decreased with the increased concentrations. A calibration curve was obtained for the increasing concentration vs. the DPV current, and it fitted with the Hill1 function [31] as given by,

(2) $$y=START+\left(END-START\right)\frac{x^{n}}{k^{n}+x^{n}}$$

where *START*, *END* is the minimum and maximum response of the sensor; *k* = Michaelis constant; *n* = Cooperative sites (Hill coefficient). The best-fit values of the experimental values are shown in inset Figure 7 and Figure 8 (bottom row). For the determination of the *y*_LOD_ = *START* + 3 × standard deviation of the blank, (SD_blank_) was chosen as it provides a more realistic LOD [32]. The LOD values for the bacteria detection using the total bacteria probe were found to be 5.8 × 10^1^, 14.6 × 10^1^, and 8.1 × 10^1^ CFU/mL for *S. Typhimurium*, *E.Coli*, and *S. aureus*, respectively. However, these values were better when the assay was performed with selective aptamer probes, with observed LODs of 4.2 × 10^1^, 6.1 × 10^1^, and 4.4 × 10^1^ CFU/mL for *S. Typhimurium*, *E.Coli*, and *S. aureus*, respectively. The linear range of the assay performed with selective probes was 10^0^ to 10^3^ CFU/mL, with the total bacteria probe possessing 10^0^ to 10^4^ CFU/mL. All the experiments at different concentrations were performed quadruplicated.

### 3.5. Selectivity, Stability, and Reproducibility of the Assay for Practical Applications

**Figure 9:**
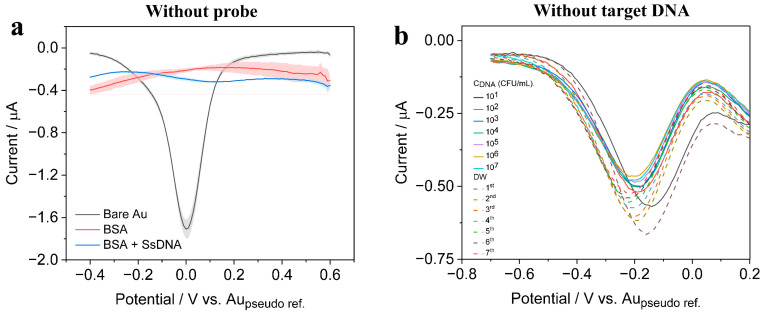
(**a**) DPV curves recorded from the aptamer-free sensor (negative control) after incubation with 1% BSA; (**b**) DPV curves observed towards the estimation of selectivity of the sensor, by adding just DI water instead of bacterial DNA concentrations prepared in DI water.

To examine the selectivity of the sensor, negative control experiments were performed without using the receptor aptamer (i.e., BSA/Au electrode). Different concentrations of ssDNA were incubated with the sensor and no measurable change in the DPV response was observed (Figure 9a). Similarly, for further evaluation, the sensor surface (i.e., BSA/Aptamer/Au electrode) was subjected to different concentrations of bacterial DNA in DI water, and the results were compared with DI water containing no bacterial DNA. The sensor was selective towards the bacterial DNA which clearly showed the change in DPV response; therefore, the sensor is selective towards pathogenic bacteria detection (Figure 9b). The stability of the experiments was tested to determine its long-term storage analysis; however, this sensor is currently in a state of “open, use, and throw” for one-time usage purposes. The reproducibility of the sensor was assessed with % relative standard deviation (% RSD) for the inter- and intra-assay of the sensor. The intra-assay precision of the sensor was examined by detecting 10^2^ CFU/mL of bacteria concentration in three replicate measurements, whereas the inter-assay precision was evaluated by measuring 10^2^ and 10^4^ CFU/mL of bacteria in three independently prepared BSA/Aptamer/Au electrodes. The intra- and inter-assay RSD was found to be 4.2% and 2.3%, respectively, thereby indicating acceptable levels of precision and reproducibility.

## 4. Conclusions

In this study, an electrochemical biosensor was utilized for the quantitative detection of target bacteria, and the results showed that under optimal reaction conditions, this method had good detection effects on the DNA of three different target bacteria. The biosensor has advantages such as short response time, simple equipment, low cost, and easy portability, and is a popular method for detecting pathogenic microorganisms. In the field of food safety, this method also has a wide range of application prospects.

As an important component of the biochemical sensor platform, the selection of biorecognition elements, transducer sensors, and signal amplification components is a key factor affecting the performance of the entire detection platform. Therefore, by improving its sensitivity, selectivity, and stability, more accurate and reliable detection can be achieved, thereby enhancing its practical application value in medical diagnosis, environmental monitoring, and food safety, among other fields. In addition, in-depth research and exploration are needed on the specificity, detection limit, anti-interference ability, reproducibility, and other aspects of the detection platform to meet the needs of different application scenarios.

## Figures and Tables

**Table 1:** The sequence information of the aptamers adapted for the selective and combined detection of bacterial pathogens is presented in the below table.

Aptamer	Thiol-Modified Sequences from 5′ to 3′
*Escherichia coli*	SH- AAA AAA CGG GAT GAT GTT CTG GGA A
*Salmonella typhimurium*	SH- AAA AAA CGA CGC CGG ATT CCC CTA CCA G
*Staphylococcus aureus*	SH- AAA AAA ATG ACC AGC TTC GGT ACT AAA GAT
*Total bacteria*	SH- AAA AAA ATT ACC GCG GCT GCT GGC

## Data Availability

All research data are included in this article.
